# AQP9 Is a Prognostic Factor for Kidney Cancer and a Promising Indicator for M2 TAM Polarization and CD8+ T-Cell Recruitment

**DOI:** 10.3389/fonc.2021.770565

**Published:** 2021-11-05

**Authors:** Jibo Jing, Jin Sun, Yuqing Wu, Nieke Zhang, Chunhui Liu, Saisai Chen, Wenchao Li, Cheng Hong, Bin Xu, Ming Chen

**Affiliations:** ^1^ Institute of Urology, Surgical Research Center, Institute of Urology, Medical School of Southeast University, Nanjing, China; ^2^ Department of Urology, Medical School of Southeast University, Nanjing, China; ^3^ Department of Urology, Affiliated Zhongda Hospital of Southeast University, Nanjing, China; ^4^ Department of Urology, People’s Hospital of Xuyi County, Nanjing, China

**Keywords:** tumor microenvironmental, renal clear cell carcinoma (KIRC), TCGA, extracellular matrix (ECM), tumor-associated macrophages (TAMs), AQP9

## Abstract

**Background:**

It is undeniable that the tumor microenvironment (TME) plays an indispensable role in the progression of kidney renal clear cell carcinoma (KIRC). However, the precise mechanism of activities in TME is still unclear.

**Methods and Results:**

Using the CIBERSORT and ESTIMATE calculation methods, the scores of the two main fractions of tumor-infiltrating immune cells (TICs) from The Cancer Genome Atlas (TCGA) database of 537 KIRC patients were calculated. Subsequently, differentially expressed genes (DEGs) were drawn out by performing an overlap between Cox regression analysis and protein–protein interaction (PPI) network. Aquaporin 9 (AQP9) was identified as a latent predictor through the process. Following research revealed that AQP9 expression was positively correlated with the pathological characteristics (TNM stage) and negatively connected with survival time. Then, by performing gene set enrichment analysis (GSEA), it can be inferred that genes with high expression level of *AQP9* were mainly enriched in immune-related activities, while low *AQP9* group was associated with functions of cellular metabolism. Further studies have shown that regulatory T cells (Tregs), macrophages M2, macrophages M0, CD4+ T cells, and neutrophils were positively correlated with *AQP9* expression. While the levels of mast cells, natural killer (NK) cells, and CD8+ T cells are negatively correlated with *AQP9*. The result of multiple immunohistochemistry (mIHC) suggests a negative relevance between AQP9 and CD8+ T cells and reveals a trend of consistent change on *AQP9* and M2 macrophages.

**Conclusion:**

The expression level of *AQP9* may be helpful in predicting the prognosis of patients with KIRC, especially to the TME state transition, the mechanism of which is possibly through lipid metabolism and P53, Janus kinase (JAK)/signal transducer and activator of transcription (STAT) pathways that affect M2 polarization. *AQP9* was associated with the expression levels of M2, tumor-associated macrophages (TAMs), and the recruitment of CD8+ T cells in tumor environment. The research result indicates that *AQP9* may be an obstacle to maintain the immune activity of TME.

## Introduction

Kidney renal clear cell carcinoma (KIRC) is the most common kidney tumor, accounting for 60%~75% of all sorts of kidney tumors (1). The mortality rate of KIRC is relatively high, and the 5-year survival rate is as low as 10%. Even if patients showed no signs of distant metastasis at the time of diagnosis, recurrent metastasis still occurred in 20%–30% of patients after surgical treatment. According to previous studies, the metastases have already existed in approximately 25%~30% patients with KIRC who turned to medical care for the first time (2, 3). Therefore, it is necessary to pay attention to early screening on potential metastasis in patients with KIRC, which may help predict clinical outcomes more accurately.

The tumor microenvironment (TME) is the inside condition of tumors where tumor cells originate and survive. The components, in addition to tumor cells themselves, comprise a variety of cells, such as fibroblasts, immune and inflammatory cells, and glial cells (4). These are collectively referred to as the extracellular matrix (ECM), which is an extremely complex molecular environment and is essential in the assembly of macroscopic three-dimensional structures. They have been reported to participate in the process of tumorigenesis and development through a variety of mechanisms (5). Immune cells and stromal cells have been reported to be important for tumor diagnosis and prognostic evaluation. ESTIMATE is precisely an algorithm for tumor purity determination based on gene expression information (6). It visually scores the degree of non-tumor cell infiltration by analyzing the expression profiles of certain immune and stromal cell-associated specific genes. The algorithm has been widely used in tumors such as pancreatic, breast, colon, and prostate cancers, as well as in immune infiltration and microenvironment-related analysis (7–10).

Although TME has been known to play an important role in the polarization and formation of tumor-associated macrophages (TAMs) (11), its mechanism is still unclear. Some studies have reported that cathelicidin-related antibacterial peptides derived from prostate cancer can impede the transformation from MΦs to TAMs, while oncostatin M and eosinophil chemokine derived from hypoxic cancer cells promote the differentiation of MΦs into TAMs (12). Most TAMs in TME present M2-like phenotypes and are involved in the modulation of tumor growth and metastasis through various pathways (13–15). The increase of TAMs is associated with worse clinical outcomes in patients with different kinds of cancers. Although a recent study showed that fatty acid oxidation (FAO) had an effect on the functional polarization of TAMs (16), it is still unclear how M2 polarization is promoted and how TAMs inhibit antitumor immunity in TME.

Obviously, amounts of dedication and investment are required to fully ascertain the specific mechanism of the TME. However, we learned from experience that no matter how complex the functional system is, it ultimately depends on the expression of specific genes for execution. Therefore, searching for these functional genes related to tumor immunity and exploring the mechanism of their interaction with TME are the first steps to solve the problem. We assume that there are kinds of genes whose expression levels are closely related to the state of the TME. Meanwhile, in many of these similar genes, some genes play more critical roles in regulating the state of the TME or functioning as the specific targets of TME. Furthermore, by analyzing the relationship between this gene and the clinical prognosis of KIRC, we can initially screen out a TME-related indicator, a potential immunotherapy target.

The aquaporin 9 (*AQP9*) is a member of the aquaporin family that acts as a water-selective membrane channel, and it may be involved in cell migration, angiogenesis, and tumor growth (17). In an earlier report, the protein translated from this gene functions as a channel that allows a variety of uncharged solutes to pass through. Then, it also functions as a stimulus of urea transport and water penetration (18). The encoded protein essentially is involved in specialized leukocyte functions such as immunological response and bactericidal activity. Also, it can facilitate the uptake of glycerol in hepatic tissue.

In this study, we compared differentially expressed genes (DEGs) between two different TME ingredients in KIRC patients by ESTIMATE package and investigated the function of *AQP9* in altered TME status.

## Results

### Research Process of Presented Study

**Figure 1:**
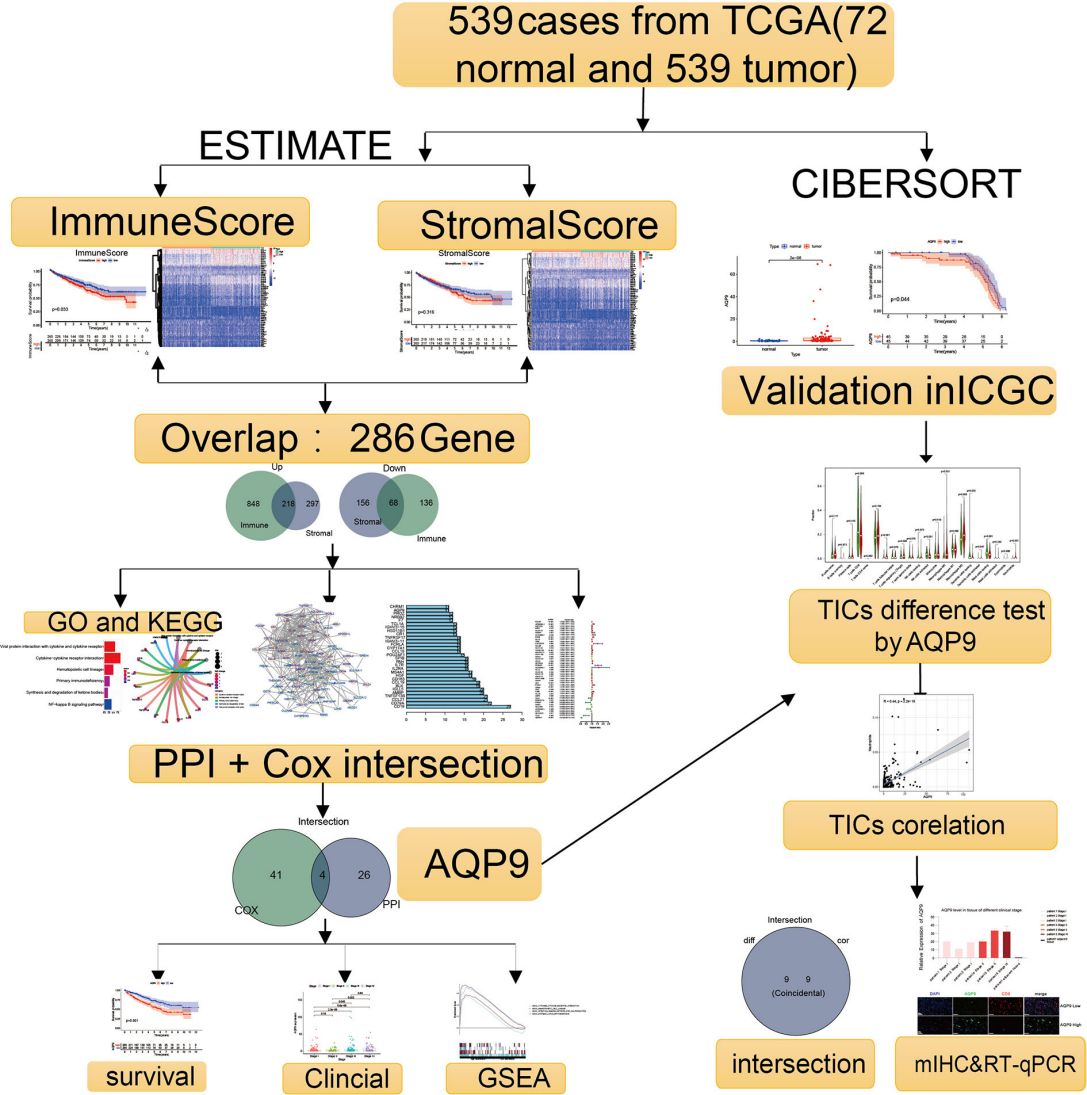
Flowchart of the research.

The analysis procedure of this study is shown in **Figure 1**. To estimate the fraction of tumor-infiltrating immune cells (TICs) and the amount of immune or matrix components in the KIRC samples, RNA sequencing (RNA-seq) data from 551 patients and corresponding clinical data of 537 cases were downloaded from The Cancer Genome Atlas (TCGA) database. The baseline data of enrolled cases are shown in **Table 1**.

**Table 1:** Baseline characteristics of enrolled cases.

Characteristics	TCGA cohort n = 537 No. of patients (%)	ICGC cohort n = 90 No. of patients (%)
Age ≤65	336 (62.57)	62 (68.89)
Male	346 (64.43)	51 (56.67)
Pathologic_T		
T1	275 (51.21)	54 (60.00)
T2	69 (12.85)	13 (14.44)
T3	182 (33.89)	21 (23.33)
T4	11 (2.05)	2 (2.22)
Tx or unknown	0	0
Pathologic_M		
M0	426 (79.33)	81 (90.00)
M1	79 (14.71)	8 (8.89)
Mx or unknown	32 (5.96)	1 (1.11)
Pathologic_N		
N0	240 (44.69)	78 (86.67)
N1	17 (3.17)	2 (2.22)
Unknown	280 (52.14)	10 (11.11)
Histologic_grade		
G1	14 (2.61)	
G2	230 (42.83)	
G3	207 (38.55)	
G4	78 (14.53)	
Gx or unknown	8 (1.49)	
Tumor_stage		
Stage I	269 (50.09)	
Stage II	57 (10.61)	
Stage III	125 (23.28)	
Stage IV	83 (15.46)	
Status		
Alive	360 (67.04)	61 (67.78)
Dead	177 (32.96)	29 (32.22)
Unknown	3 (0.56)	0
Follow-up time		
t ≤365	97 (18.06)	7 (7.78)
1,825 ≥ t > 365	290 (54.00)	42 (46.67)
t >1,825	150 (27.93)	41 (45.56)

ICGC, International Cancer Genome Consortium; TCGA, The Cancer Genome Atlas.

Using CIBERSORT and ESTIMATE calculation methods, the scores of the two main fractions of TICs were calculated. Subsequently, DEGs were drawn out by performing an overlap between Cox regression analysis and protein–protein interaction (PPI) network. *AQP9* was identified as a latent predictor through the process. In subsequent analyses, the prognostic value of *AQP9* was verified by survival analysis, differential expression, etc. Then, gene set enrichment analysis (GSEA) and correlation with TICs were performed to explore the potential mechanism. Finally, we conducted the preliminary verification through multiple immunohistochemistry (mIHC) and real-time quantitative PCR (RT-qPCR).

### Survival Correlation

Kaplan–Meier survival analysis was adopted for the calculation of ImmuneScore, StromalScore, and ESTIMATEScore in order to figure out the relationship between estimated ratio and patients’ survival duration. Higher estimated scores indicate a larger number of corresponding components in the TME. ESTIMATEScore is the sum of the two ingredients and represents the comprehensive percentage of all immune-related ingredients.

**Figure 2:**
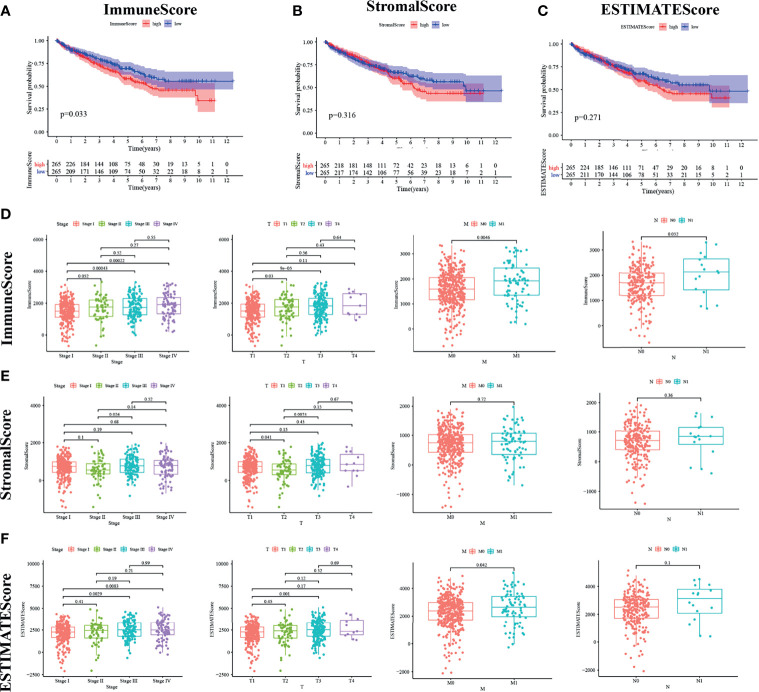
ImmuneScore is correlated with survival of kidney renal clear cell carcinoma (KIRC) patients and clinical TNM staging. **(A–C)** According to the ESTIMATE algorithm, survival analysis of high and low ImmuneScore, StromalScore, and ESTIMATEScore. Panels **(D–F)** show the bar plot of the three scores with tumor clinical staging and TNM staging, respectively.

As shown in **Figure 2A**, the immune components present a negative correlation with the overall survival rate of KIRC patients. However, the other components had no significant correlation with the overall survival rate (**Figures 2B, C** **)**. These results implied that the immune components in TME were more suitable for indicating the prognosis of KIRC patients.

### Pathological Stage and TNM Classification Were Associated With the Calculated ImmuneScore

We analyzed the clinical information of KIRC patients in order to clarify the relationship between the percentage of immune and stromal components and pathological properties. As shown in **Figure 2**, ImmuneScore was positively correlated with M and N classification of TNM stage (**Figure 2D**). StromalScore was not significantly correlated with TNM stage (**Figure 2E**), whereas ESTIMATEScore was strongly related with increasing N stage (**Figure 2F**; p = 0.042). It can be inferred from these results that the elevated proportion of the immune component was strongly associated with KIRC progression such as invasion and metastasis of tumor.

### Enrichment Analysis on Differentially Expressed Genes Presents a Possible Relation With Immune Function

**Figure 3:**
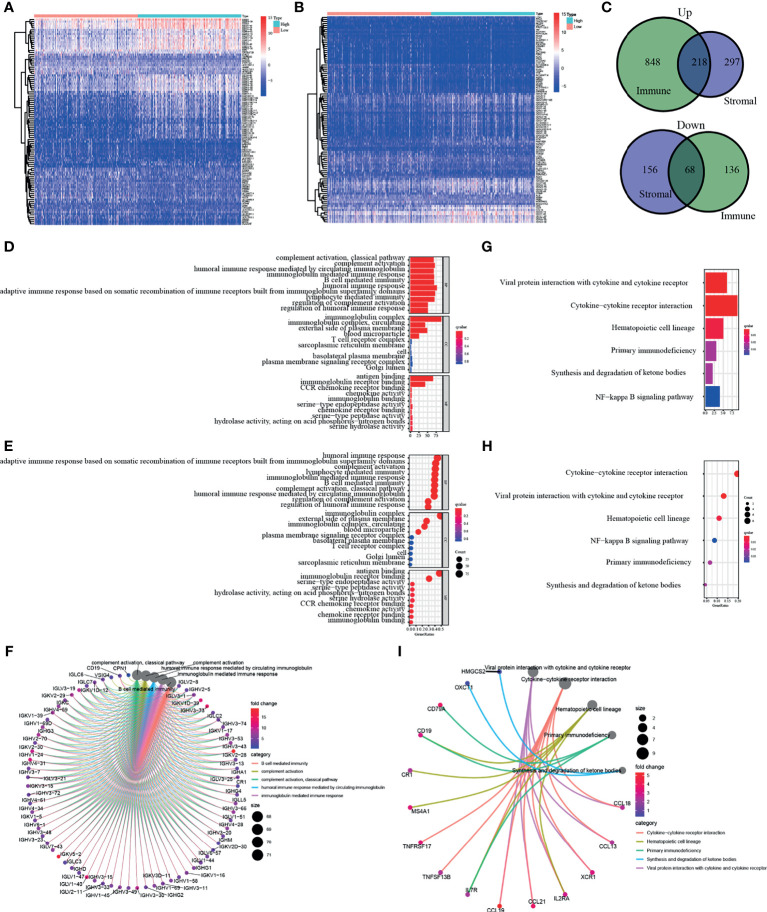
Enrichment analysis of differentially expressed genes (DEGs). DEGs of two immune-related ingredients in tumor microenvironment (TME) were shown in panels **(A, B).** In panel **(C)**, a total of 286 genes shared by the ImmuneScore and StromalScore. **(D–F)** Result of Gene Ontology (GO) enrichment analysis. **(G–I)** Kyoto Encyclopedia of Genes and Genomes (KEGG) enrichment analysis of DEGs.

In order to elucidate the clear changes in gene distribution related to two immune-related ingredients in TME, the DEGs were divided into high and low score groups, and comparative analysis was performed subsequently. The result of intersection is shown in the Venn diagram, and it turns out that a total of 218 upregulated genes were shared by the ImmuneScore and StromalScore, as well as 68 downregulated genes shared by the low score group. These DEGs (a total of 286 genes) may be decisive factors of TME status (**Figures 3A–C**). It is shown by the results of Gene Ontology (GO) enrichment analysis that almost all DEGs were enriched in the functions related to immunity, such as humoral immunity and complement system (**Figures 3D–F**). Kyoto Encyclopedia of Genes and Genomes (KEGG) enrichment analysis also showed enrichment for primary immunodeficiency, cytokine–cytokine receptor interactions, and hematopoietic cell profiles (**Figures 3G–I**). Thus, the overall function of DEGs appeared to be related to immune-associated activities, suggesting that the presence of immune factors was a distinguishing part of TME in KIRC.

### Protein–Protein Interaction Network and Univariate Cox Regression

**Figure 4:**
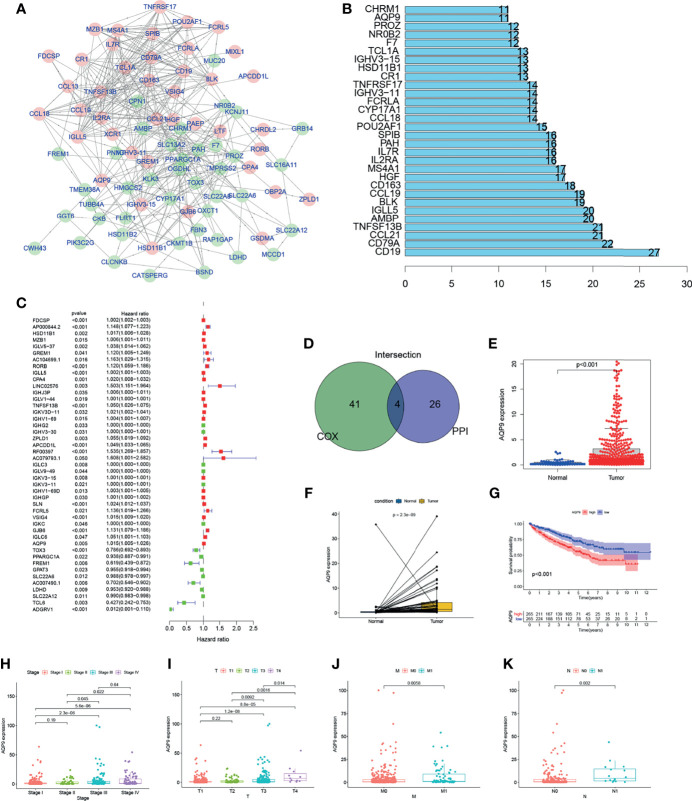
The screening process and initial validation in clinical information of aquaporin 9 (AQP9). Genes with the top 20 Interaction node number **(B)** according to protein–protein interaction (PPI) network **(A)** were screened out. **(D)** Overlap between the uni-Cox regression **(C)** and PPI network. Expression bar chart **(E)**, paired expression bar chart **(F)**, and survival analysis **(G)** of AQP9. **(H–K)** The expression level of AQP9 in kidney renal clear cell carcinoma (KIRC) patients with different clinical and TNM stages.

Further explorations focus on the detailed mechanisms of those genes in KIRC. PPI network was performed using Cytoscape software [National Institute of General Medical Sciences (NIGMS)] based on the STRING (Search Tool for the Retrieval of Interacting Genes/Proteins) database. Interaction results of 286 genes are shown in **Figure 4A**, and the top 30 genes with the largest number of nodes in PPI network have been listed in the bar plot (**Figure 4B**). Uni-COX regression was subsequently performed to find out which gene is more important among the 286 DEGs (**Figure 4C**). Then, an overlap between the PPI network and uni-Cox regression was performed, and only four genes (*AQP9, TNFRSF13B, IGLL5, HSD11B1*) were presented eventually (**Figures 4A–D**).

### The Correlation of AQP9 Expression With Survival and Clinical Characteristics

Among the four candidates, TNF Superfamily Member 13b *TNFRSF13B* is known as a member of the TNF Superfamily, which proved to induce apoptosis by interacting with other members of TNF receptor family (19, 20). Also, studies have suggested that Immunoglobulin Lambda Like Polypeptide 5 *IGLL5* may be involved in the regulation of TME (21). However, we believe such influence of Immunoglobulin Lambda Like Polypeptide 5 comes from a deeper layer, since it encodes the lambda polypeptides of immunoglobulin, which are often extensive and nonspecific. Therefore, we believe that this kind of connection is meaningless. As for Hydroxysteroid 11-Beta Dehydrogenase 1 *HSD11B1*, its translation product is a catalyst for the generation of corticosteroids. Current research believes that its function is mainly concentrated in endocrine (22, 23).

*AQP9*, which is an aquaporin, is believed to mainly focus on metabolism and white blood cell functions (18). The changes in the number of CD8+ T cells in the immune microenvironment are particularly related to lipid metabolism, and *AQP9* happens to be connected with the regulation of this aspect (24, 25). Therefore, we chose *AQP9* for further research.

In this study, all KIRC samples were divided into two groups with high or low *AQP9* expression. The conclusion that the survival duration of KIRC patients with high *AQP9* expression is shorter than that in the low expression group can be obtained from survival analysis (**Figure 4G**). Furthermore, it can be inferred from the results of Wilcoxon rank sum test that the expression of *AQP9* in tumor samples is significantly higher than that in normal samples (**Figure 4E**). Similar results were observed in the paired analysis between normal and tumor tissue from the same patient (**Figure 4F**). The above results clearly show that the expression of *AQP9* in TME was negatively correlated with survival outcomes. In particular, the expression of *AQP9* increased with the progression of the TNM phase (**Figures 4H–K**).

**Figure 5:**
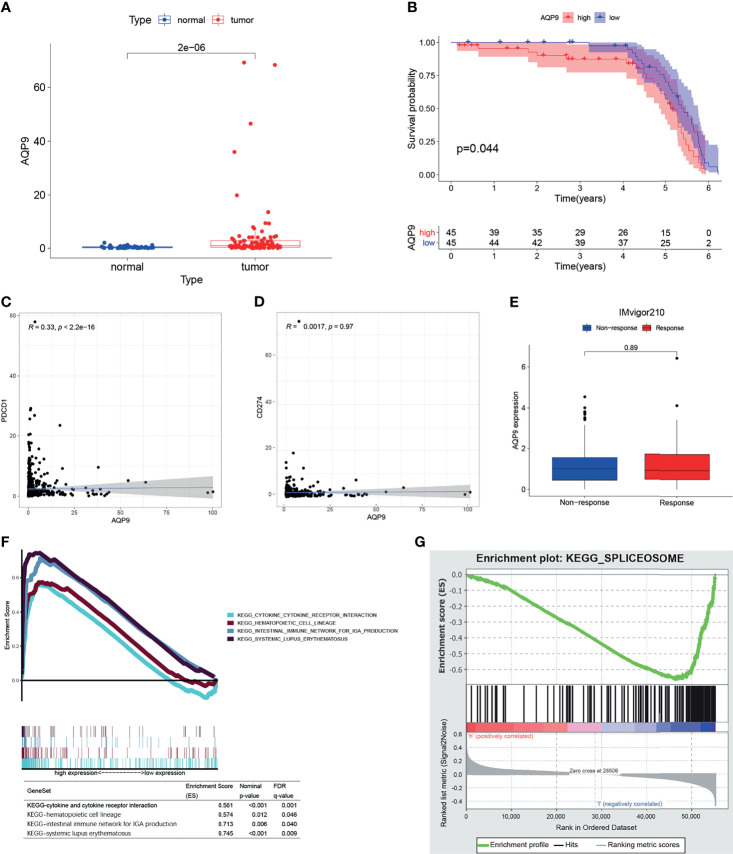
**(A)** The differential expression of AQP9 in International Cancer Genome Consortium (ICGC) tumors and adjacent tissues. **(B)** Survival analysis from of data form ICGC cohort. **(C, D)** Correlation plot of aquaporin 9 (AQP9) to programmed cell death-1 (PD-1) and AQP9 to programmed cell death-ligand 1 (PD-L1). **(E)** AQP9 predicting immune response by IMvigor210. **(F, G)** Result of gene set enrichment analysis (GSEA).

Furthermore, we downloaded the clinical data (**Table 1**) and RNA-seq data of 90 KIRC patients from the International Cancer Genome Consortium (ICGC) database (including tissues of 90 primary tumors and 46 adjacent tissues) in order to verify the results above. Consistent with the results of TCGA, *AQP9* was significantly higher in tumor tissues (p < 0.001) (**Figure 5A**), and higher *AQP9* levels were associated with shorter overall survival duration (p = 0.044) (**Figure 5B**).

### *AQP9* Has Potential to Be an Indicator of Tumor Microenvironment Modulation

GSEA results are applicable to both the high and the low expression groups because the *AQP9* level is positively correlated with survival and TNM stage. As shown in **Figure 5F**, the genes in the high expression group of *AQP9* present an association with immune-related activities [such as P53, Janus kinase (JAK)/signal transducer and activator of transcription (STAT), nod-like receptor signaling pathway, cytokine and its receptor interaction]. As for the low-*AQP9* group, an apparent enrichment of these genes can be observed in fatty acid metabolism pathways (**Figure 5G**). Spliceosome is a complex composed of ribonucleoprotein (RNP) and protein, which is involved in the occurrence and development of various diseases by functioning as stimulus for the splicing of pre-mRNA (26). On the one hand, it has been reported that the spliceosome is involved in the regulation of some key genes in lipid metabolism. For instance, removal of exon 9 in fatty acid synthase (FASN) by splicing significantly reduced the production of the synthesis of fatty acid (27). On the other hand, as an aquaporin, *AQP9* is indeed very likely to participate in the transport of fatty acids. Some previous studies have emphasized the relationship between fatty acid metabolism and polarization of TME (28). Therefore, it can be drawn from these results that *AQP9* may be a potential indicator of TME status.

### AQP9 Has Little Value in Predicting Immunotherapeutic Benefits

Immunotherapy, which is used in cancer treatment to block the immune checkpoints such as programmed cell death-1 (PD-1) and programmed cell death-ligand 1 (PD-L1), has shown promising outcomes in advanced cancers. However, it is not effective in all patients. In the subsequent analysis, we performed correlation analysis between *AQP9* and PD-1, PD-L1 to clarify if *AQP9* is able to predict the benefits of immunotherapy for patients. Furthermore, the patients who received anti-PD-L1 immunotherapy in the IMvigor210 cohort were assigned to response or non-response group, and expressions of *AQP9* between the two groups were compared. However, none of these analyses indicated that *AQP9* could be correlated to response to immunotherapy (**Figures 5C–E**).

### Correlation of *AQP9* With Immune Cells

**Figure 6:**
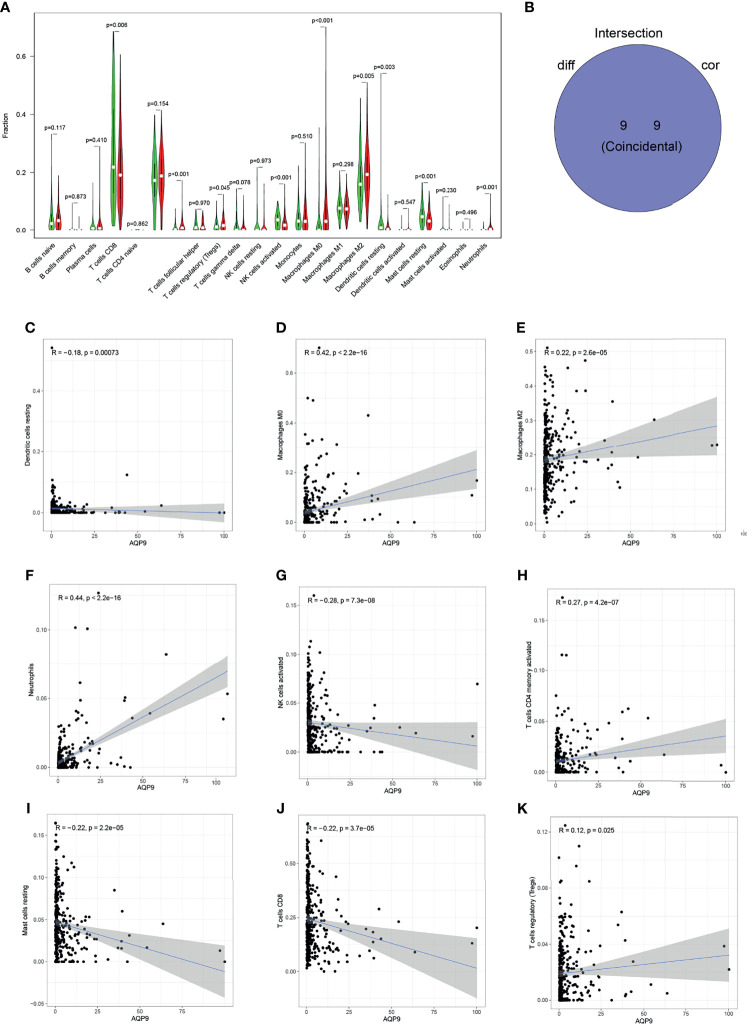
Relationship between aquaporin 9 (AQP9) and immune cells. **(A)** The differential expression of various types of immune cells in cancer and adjacent tissues. **(B)** Overlap of correlation and differential analysis of AQP9. **(C–K)** Correlation analysis of the expression of AQP9 and the level of different immune cells.

The proportion of tumor-infiltrating immune-related subgroups was obtained by using the CIBERSORT algorithm. Therefore, the relationship between *AQP9* and the immune microenvironment can be further derived, and 21 immune cell profiles were also constructed in KIRC samples. Among them, there were 11 kinds of immune cells presenting obviously different expressions between tumor and adjacent tissues (**Figure 6A**). Then, immune cell correlation was performed, and a total of nine immune cells were shown to be related to the expression of *AQP9*. After the intersection between differentially expressed immune cells and related cells, nine immune cells related to the TME were finally obtained (**Figure 6B**). Five of the immune cells were positively correlated with *AQP9* expression, including regulatory T cells (Tregs), M2 macrophages, M0 macrophages, CD4 T cells, and neutrophils, while resting mast cells, resting natural killer (NK) cells, and CD8 T cells were negatively correlated with *AQP9* levels. These results served as strong evidence to further confirm that *AQP9* was involved in the regulation of TME immune reactivity (**Figures 6C–K**).

### The Expression of *AQP9* Increased Correspondingly With the Increase of Tumor Stage Verified by RT-qPCR

**Figure 7:**
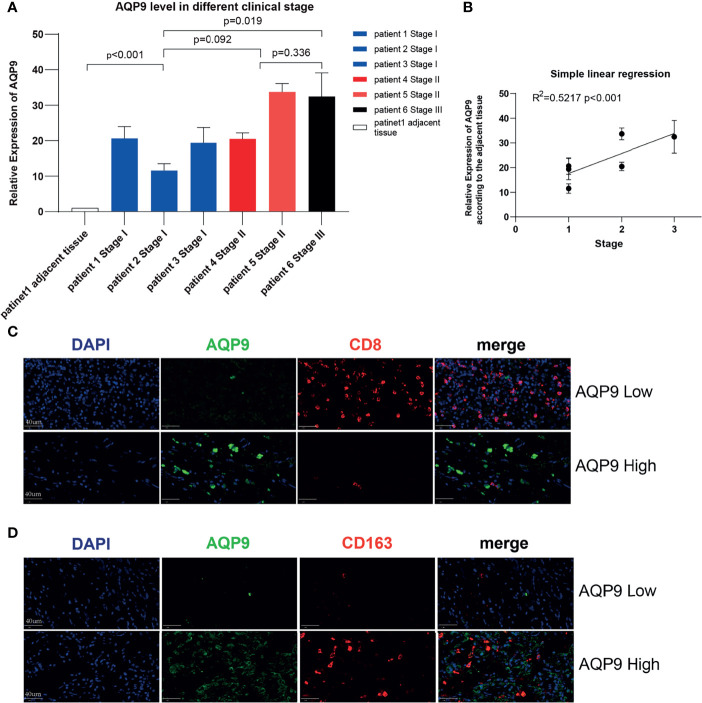
Validation in kidney renal clear cell carcinoma (KIRC) patients. **(A)** The real-time quantitative PCR (RT-qPCR) result of aquaporin 9 (AQP9) in patients of different KIRC stages. **(B)** Simple linear regression of AQP9 level according to the different stages. **(C, D)** Multiple immunohistochemistry (mIHC) result of AQP9, CD163, and CD8 in KIRC tumor tissue.

In order to further verify the potential correlation between the *AQP9* level and tumor stage mentioned above, we applied this on six KIRC samples of different stages and a para-cancerous sample from the sample bank (**Table 2**). Total RNA expression was extracted from these samples for RT-qPCR. As shown in **Figure 7A**, *AQP9* expression was relatively higher in high-stage KIRC specimens. In the subsequent analysis, a simple linear regression was performed on the *AQP9* levels of different stages, and the results verified that the *AQP9* expression level was positively correlated with the tumor stage (R^2^ = 0.5217, p < 0.001) (**Figure 7B**).

**Table 2:** General information of clinical samples.

Tumor							
Patients	Gender	Age	Tumor size (cm)	Location of tumor	Stage	TNM stage	Pathological type
Patient 1	Male	64	4.7 × 4.3 × 4.5	Left	Stage I	T1aN0M0	KIRC
Patient 2	Male	59	3.5 × 4.5 × 2.5	Upper right		T1bNxM0	KIRC
Patient 3	Female	48	3.2 × 3.5 × 3.3	Right		T1aNxMx	KIRC
Patient 4	Male	69	2 × 2 × 2	Right	Stage II	T2aN0M0	KIRC
Patient 5	Female	47	5×4×1.5	lower left		T2bNxMx	KIRC
Patient 6	Male	72	7.5×6×6.5	left	Stage III	T3N1M0	KIRC

The clinical features of the six kidney renal clear cell carcinoma (KIRC) patients.

### Results of mIHC Confirmed That the Expression Status of *AQP9* Is Correlated With the Levels of CD8+ T Cells and M2 Tumor-Associated Macrophages

Since the potential connection between *AQP9* and immune cells in the TME may be the key to its function, mIHC was performed on pathological specimens of renal clear cell carcinoma in order to further verify the possible relevance in clinical samples. It was observed in the tumor center that when the tumor tissue presented a high *AQP9* level, the infiltration of CD8+ T cells was significantly blocked. Conversely, the recruitment of CD8+ T cells was relatively active in areas with low *AQP9* level (**Figure 7B**). In addition, consistent with the above results, the quantity of M2 TAMs (labeled by CD163) and *AQP9* showed obvious consistency. Therefore, the active *AQP9* expression may be deeply involved in the polarization process of M2 TAMs (**Figures 7C, D**). These results preliminarily verified our inferences in the bioinformatics analysis that *AQP9* was indeed related to M2 TAM levels and CD8+ T-cell recruitment.

## Discussion

In the beginning, we aimed to search for genes from TCGA database that are related to TME and can help predict the clinical characteristics of KIRC patients. Finally, *AQP9* was chosen, and after a series of bioinformatics analyses, it was considered to be a latent modulator of TME activity.

It is now widely acknowledged that the TME plays a crucial role as a starting point for cancer treatment. To find and explore the mechanism of TME in specific tumors and to change the status of the TME in tumors, new therapeutic targets are needed in the future (4). Conclusion that the immune component in TME helps to improve the clinical outcomes of patients is shown by the results of transcriptome analysis. In particular, inference that the invasion and metastasis of KIRC have a significant linear relationship with ESTIMATEscore also could be drawn. These results indicate the possible conditions in tumor immunity through a series of effective bio-analysis methods and provide a possible incentive for tumor treatment.

Although there are many available options for treatment, surgery remains the benchmark for the treatment of localized clear cell carcinoma. However, there are still certain limitations of surgical treatment, such as unresectable lesions, relapse, and metastasis of tumors after operation (29). Scosyrev et al. (30) found that stable renal insufficiency has long been observed in patients with normal renal function, most of which have surgery-related low GFR. In addition, it is unfeasible to prove that elderly patients have a longer survival time when comparing surgery with non-surgical management (31). In recent years, immunotherapy has emerged rapidly, and there have been a lot of research in the basic field. Therefore, it is a thorny issue that the existing immunotherapy methods have defects such as adverse reactions and low sensitivity toward KIRC. Research in this field on exploration of new possible solutions has become particularly important.

Furthermore, our analysis based on the RNA-seq data found that the expression of *AQP9* was closely related to clinical features such as staging and distant metastasis as well as poor prognosis. Therefore, we can infer that *AQP9* is an official target that may play an important role in TME and is of great significance.

*AQP9* is a member of the aquaporin family that acts as water-selective membrane channel. This structure is reported to play a key role in immunological response and bactericidal activity. Coincidentally, it also has been reported that *AQP9* may be involved in cell migration, angiogenesis, and tumor growth (17).

It is shown that *AQP9* tends to express more as the stage increases, which is unanimous with our linear correlation results of TME-related immune cells. Thus, we can speculate that *AQP9* may play a role in not only reducing survival time in tumors but also suppressing the tumor immunity. Besides, it has been reported that *AQP9* might be involved in immunological response. Therefore, the connection between the expression level of *AQP9* in tumor cell and TME was researched subsequently. According to the results of GSEA, the signal pathways associated with immunity were significantly enriched in the high-*AQP9* group, such as P53, JAK/STAT, nod-like receptor signaling pathway, and cytokine and its receptor interaction. In the low-*AQP9* group, the enrichment in fatty acid metabolism pathway was remarkably higher. It can be assumed that *AQP9* might get involved in tumor immunity by inducing the conversion of TME into an immunosuppressive state, and fatty acid metabolism may take part in this conversion. This conclusion is consistent with the following research on the proportion of immune cells. Accordingly, the cancer-promoting effects and the changes of components in the immune microenvironment are related to the high expression of *AQP9*, and TME transformations all suggest that *AQP9* is related to the malignant degree of KIRC.

The JAK/STAT pathway is one of the few signaling pathways involved in a variety of independent biochemical processes in cells, serving as the main way of multiple signal transductions inside and outside the cell (32).

JAK was also reported previously to mediate the recruitment of other molecules such as mitogen-activated protein (MAP) kinase and phosphoinositide (PI)3 kinase, in addition to the function in activating STAT. The Ras-Raf-MAP kinase and PI3 kinase pathways act as downstream signal processors for these molecules, thereby mediating a series of pretranscriptional regulation (33). It has been reported that JAK inhibition could reprogram the TME toward an anti-inflammatory/antitumorigenic status in lung adenocarcinoma (34). The present research shows that *AQP9* may take part in the transaction of TME toward an antitumorigenic status in KIRC through JAK/STAT pathway.

Many external signal stimuli can induce the activation of P53. This signal includes DNA damage, oxidative stress, and the activation of proto-oncogenes. P53 mainly acts as a special transcription factor after transcription, which produces three main outputs, including cell cycle arrest, cell senescence, or apoptosis (35). Maintaining P53 levels in TME is related not only to a significant increase in macrophage infiltration into tumors but also to M1 polarization. The reduction of P53 effectively reversed the immunosuppressive status of TME (36). Although *AQP9*-mediated chemoresistance of human melanoma downregulated the expression of apoptosis genes P53 and Bax (37), which is consistent with our study, *AQP9* is positively correlated with the increase in M2 polarization and may exert its tumor suppressor effect through the P53 pathway according to our results.

The most common type of TAMs is an anti-inflammatory M2-like phenotype, which promotes tumor growth in normal cells. However, the questions of how the macrophages stimulated the polarization to M2 and how the polarized M2 macrophages can exert its antitumor effect in TME remain to be studied. In the present study, *AQP9* was associated with not only increased M2 level but also damped CD8+ T-cell level. It is early reported that the function of chemotactic cytokines (such as *C-X-C Motif Chemokine Ligand 9 (CXCL9)* and *C-X-C Motif Chemokine Ligand 10 (CXCL10)*) was inhibited by M2, thereby inhibiting the recruitment of CD8+ T cells in TME (38). Recruitment and activation of NK cells are also linked to the switch of M2 macrophage phenotype to pro-inflammatory M1 and acted as a nonspecific antitumor role in lung cancer (39). Therefore, we speculate that the interaction between *AQP9* and M2 can hinder the recruitment of CD8 T cells and NK cells and ultimately push TME to a tumor-friendly direction.

Tregs are essential for the stability of the immune environment. Tumor cells promote the tumor immune escape by tricking Tregs, and it is speculated that this effect is implemented by promoting inhibitory TME. It has been reported that enhancing lipid accumulation and its FAO is necessary for the differentiation and activation of M2-like TAMs (40). In addition, another study found that Tregs can inhibit the secretion of interferon-γ (IFNγ) from CD8+ T cells, which would eventually prevent fatty acid synthesis by activating several specific targets (41) and ultimately selectively maintain the metabolic adaptability of M2-like TAMs.

Taking all above together, we could propose a model that *AQP9* promotes M2-like TAM polarization through Treg-regulated lipid metabolism and P53, JAK/STAT pathways, thereby inhibiting the recruitment of NK and CD8+ T cells, and ultimately stimulates the reorientation of TME in KIRC to a tumor-friendly direction. This model explains the tumor-promoting effect of *AQP9*, and we aim to further verify it in future studies.

Currently, there are different kinds of methods to quantitatively analyze immune cells, among which CIBERSORT and xCEll are most widely used. The feature of CIBERSORT is to calculate the content of immune cells relative to total immune cells, which is mainly conducted within the dataset. The core algorithm of it is *ν-support vector regressioner* (42). While xCEll targets at a wider range of cell types and it can be conducted among samples, *ssGSEA* is the core algorithm adopted by xCEll (43). Compared with xCEll, the algorithm used by CIBERSORT is more suitable for our research because we also adopt GSEA analysis in enrichment analysis (44). The advantage of xCEll is that it can classify 64 types of immune cells, but the 22 kinds of TICs provided by CIBERSORT are enough to support the research for us.

We have noticed that some studies have also paid attention to the TME of kidney cancer, and some of them have achieved interesting progress. However, there are some shortcomings. First, most of these studies focus on establishing predictive models. They stressed more on predicting prognosis by the model and lack further exploration of how the genes in the model interact with each other (45).

At the same time, the application value and extensibility are relatively limited. Studies have emphasized the relationship between a single gene and various immune cells in TME of KIRC, but this relationship has not been verified in basic experiments or clinical specimens and has not been further explored (46, 47). For example, Xu et al. (48) also researched the prognostic value of *AQP9*, mainly in the relationship between its expression level and clinical prognosis.

Enlightenment was provided by the research above. Frankly speaking, many genes have potential prognostic value, but the more in-depth mechanism and clinical significance need to be further explored. Current bioinformatics analysis on the immune microenvironment of kidney cancer lacks further application of the results obtained. Our research attaches more importance to the interaction between *AQP9* and immune cells in TME, exploring the possible mechanism and performing preliminary verification. It is not just a list of the changes of many immune cells related to *AQP9*, and the present study is also the first one to explore the specific pattern of such change. In this study, we clarified the relationship between *AQP9*, M2, and CD8+ T cells through mIHC of clinical sample and initially proposed possible ways of how they interact with each other.

In summary, ESTIMATE algorithm was employed to dig into the TME-associated genes in KIRC through a series of analyses. *AQP9* is a latent prognostic gene for KIRC patients. More importantly, *AQP9* may play a key role in TME state transition possibly through lipid metabolism and P53, JAK/STAT pathways that affect M2 polarization. Furthermore, by performing mIHC, we preliminarily confirmed that *AQP9* presents a relevance with the expression levels of M2 TAM and the recruitment of CD8+ T cells in tumor environment. Thus, studies on clarifying the relationship between *AQP9* levels and KIRC cells *in vivo* and *in vitro* should be conducted in future research.

## Materials and Methods

### Statistical Analysis

All statistical analyses and graphing were performed by the R software (version 4.0.2) or GraphPad Prism (version 8.0.1). The Student’s t-test was used to compare the expressive difference of genes. The Spearman correlation analysis was used to evaluate the relationships between *AQP9* level and the expression of various immune-related cells. Additionally, the survival analysis was based on the Kaplan–Meier method. A p-value <0.05 was considered significant.

### Raw Data

RNA-seq data of 551 KIRC cases (normal samples, 54 cases; tumor samples, 497 cases) and corresponding data on clinical characteristics of 537 cases were downloaded from TCGA database, too (https://portal.gdc.cancer.gov/).

Clinical data (**Table 1**) and RNA-seq data of 90 KIRC patients were downloaded from the ICGC database (including tissues of 90 primary tumors and 46 adjacent tissues), and project RECA-EU samples are limited to primary tumor (https://dcc.icgc.org/).

### Generation of ImmuneScore, StromalScore, and ESTIMATEScore

The ratio of the immune matrix components of each sample in TME has been expressed in the form of three scores: ImmuneScore, StromalScore, and ESTIMATEScore, which were achieved by the ESTIMATE algorithm (R language version 4.0.2). The higher the scores are, the larger the proportions of the corresponding components are in TME.

### Survival Analysis

The Survminer package in R language is used to perform survival analysis. Among 497 cases, 458 tumor samples had detailed survival information and were capable of being included in the survival analysis. The survival curve was drawn by the Kaplan–Meier method, and the log rank was used as the statistical significance test. p < 0.05 was considered significant.

### Generation of Differentially Expressed Genes

According to the median of ImmuneScore and StromalScore, 497 tumor samples were divided into high and low groups. Difference analysis is performed using the limma package, and DEG is generated by comparing high- and low-score samples. DEGs with fold change larger than 1 after transformation of log2 (high-score group/low-score group) and false discovery rate (FDR) <0.05 were considered significant.

### Enrichment Analysis

GO and KEGG enrichment analyses using DEGs were performed by R language with the aid of clusterProfiler, enrichplot, and ggplot2 packages. p-values <0.05 were considered significantly enriched.

### Heatmaps

Heatmaps of DEGs were produced by R language with package pheatmap.

### Difference Analysis of Scores With Clinical Stages

The clinical data corresponding to the KIRC sample were downloaded from TCGA. Wilcoxon rank sum or Kruskal–Wallis rank sum test is used as significance test according to the number of clinical stages compared in the R software.

### Construction of Protein–Protein Interaction Network

Cytoscape version 3.8.1 was used to rebuild the PPI network that was constructed from the STRING database. Only nodes with a confidence level greater than 0.95 were included in the construction of the network.

### Cox Regression Analysis

R with package survival was used for univariate Cox regression. The order of p-values in univariate Cox is the main basis for gene sequencing.

### Gene Set Enrichment Analysis

GSEA was performed using the software gsea-4.1.0 (downloaded from Broad Institute), and Hallmark was used as target sets (downloaded from Molecular Signatures Database). All samples were included, and gene sets with NOM p < 0.05 were considered significant.

### Gene Expression Data With Immunotherapy

Correlation plots of *AQP9* with the target checkpoint of immune therapy (PD-1 and PD-L1) were drawn according to the data from TCGA referred above.

IMvigor210 was performed to determine the predictive value of the *AQP9*. The IMvigor210 dataset was downloaded from http://research-pub.gene.com/IMvigor210CoreBiologies. A total of 298 urothelial cancer cases with complete clinical information were enrolled.

### Tumor-Infiltrating Immune Cell Profile

The CIBERSORT calculation method was adopted to estimate the TIC abundance distribution in all tumor samples, followed by quality filtering, and only samples with p < 0.05 were included in the analysis.

The datasets are available in TCGA database (https://portal.gdc.cancer.gov).

### Real-Time Quantitative PCR

Total RNA was extracted using with EZNA Total RNA Kit (Omega Bio-tek) from six tumors and one para-carcinoma normal sample from the Sample Bank of Zhongda Hospital Affiliated to Southeast University. Primers were diluted in ddH_2_O with SYBR Green PCR Master Mix (Applied Biosystems, Japan). Running platform is StepOne Plus Real-time PCR system (Applied Biosystems, Foster City, CA, USA). Transcriptional expression was determined as the fold change of *AQP9* relative to *Glyceraldehyde-3-Phosphate Dehydrogenase (GAPDH)*. Primer sequences for AQP9 and *GAPDH* are as follows:

*AQP9*: F 5′-GAAGAGCAGCTTAGCGAAAGA-3′; R 5′-ACAGCCACATCCAAGGACAAT-3′.
*GAPDH*: F 5′-CAAGATCATCAGCAATGCCTCC-3’; R 5‘-GCCATCACGCCACAGTTTCC-3’.

The relative expression of *AQP9* mRNA in KIRC was represented as 2^-*ΔΔ*Ct  = 2^-  (*Δ*Ct (*AQP9*tumor) −  ΔCt (*AQP9*pan) , and a bar graph was drawn accordingly.

### Immunohistochemistry and Multiple Immunohistochemistry

KIRC tissues were stained by brightfield and mIHC. *AQP9* rabbit antibody was purchased from Abcam, catalog number ab8428. We used 4-μm tissue sections for both brightfield and fluorescence multiplex IHC. For brightfield, tissue sections were dewaxed and incubated in an autoclave for 5 min at 121°C in Tris-EDTA pH 7.8 antigen retrieval solution prior to blocking of endogenous peroxidase and incubation of the antibody. For multiplex fluorescence IHC, antibody staining was performed in subsequent steps including peroxidase blocking, application of the primary antibody, detection with a secondary horseradish peroxidase (HRP)-conjugated antibody, fluorescence dye detection, and microwave treatment to remove the bound antibodies. Three circulations to stain three different antibodies were performed in each experiment. Slides were subsequently counterstained with diamidino-2-phenylindole (DAPI) and mounted in antifade solution.

## Data Availability Statement

Publicly available datasets were analyzed in this study. These data can be found here: https://portal.gdc.cancer.gov/.

## Ethics Statement

The studies involving human participants were reviewed and approved by the Ethics Committee of Zhongda Hospital Affiliated to Southeast University. Written informed consent for participation was not required for this study in accordance with the national legislation and the institutional requirements.

## Author Contributions

Conceptualization, JJ and CH. Data curation, NZ. Formal analysis, JJ. Funding acquisition, BX and MC. Investigation, YW and MC. Project administration, WL. Resources, NZ. Software, JJ. Supervision, CH. Validation, YW. Visualization, YW and WL. Writing—original draft, JJ. Writing—review and editing, JJ, NZ, BX, and MC. All authors contributed to the article and approved the submitted version.

## Funding

This work was supported by the National Natural Science Foundation of China, Natural Science Foundation of Jiangsu Province (BK20161434, BL2013032, BK20150642, and BK2012336), Six Talent Peaks Project in Jiangsu Province, and Jiangsu Provincial Medical Youth Talent.

## Conflict of Interest

The authors declare that the research was conducted in the absence of any commercial or financial relationships that could be construed as a potential conflict of interest.

## Publisher’s Note

All claims expressed in this article are solely those of the authors and do not necessarily represent those of their affiliated organizations, or those of the publisher, the editors and the reviewers. Any product that may be evaluated in this article, or claim that may be made by its manufacturer, is not guaranteed or endorsed by the publisher.
