# Supplementary figures and images for: AQP9 Is a Prognostic Factor for Kidney Cancer and a Promising Indicator for M2 TAM Polarization and CD8+ T-Cell Recruitment
Source: Front Oncol. 2021 Nov 5;11:770565. doi: 10.3389/fonc.2021.770565 (PMC8602816; doi:10.3389/fonc.2021.770565)

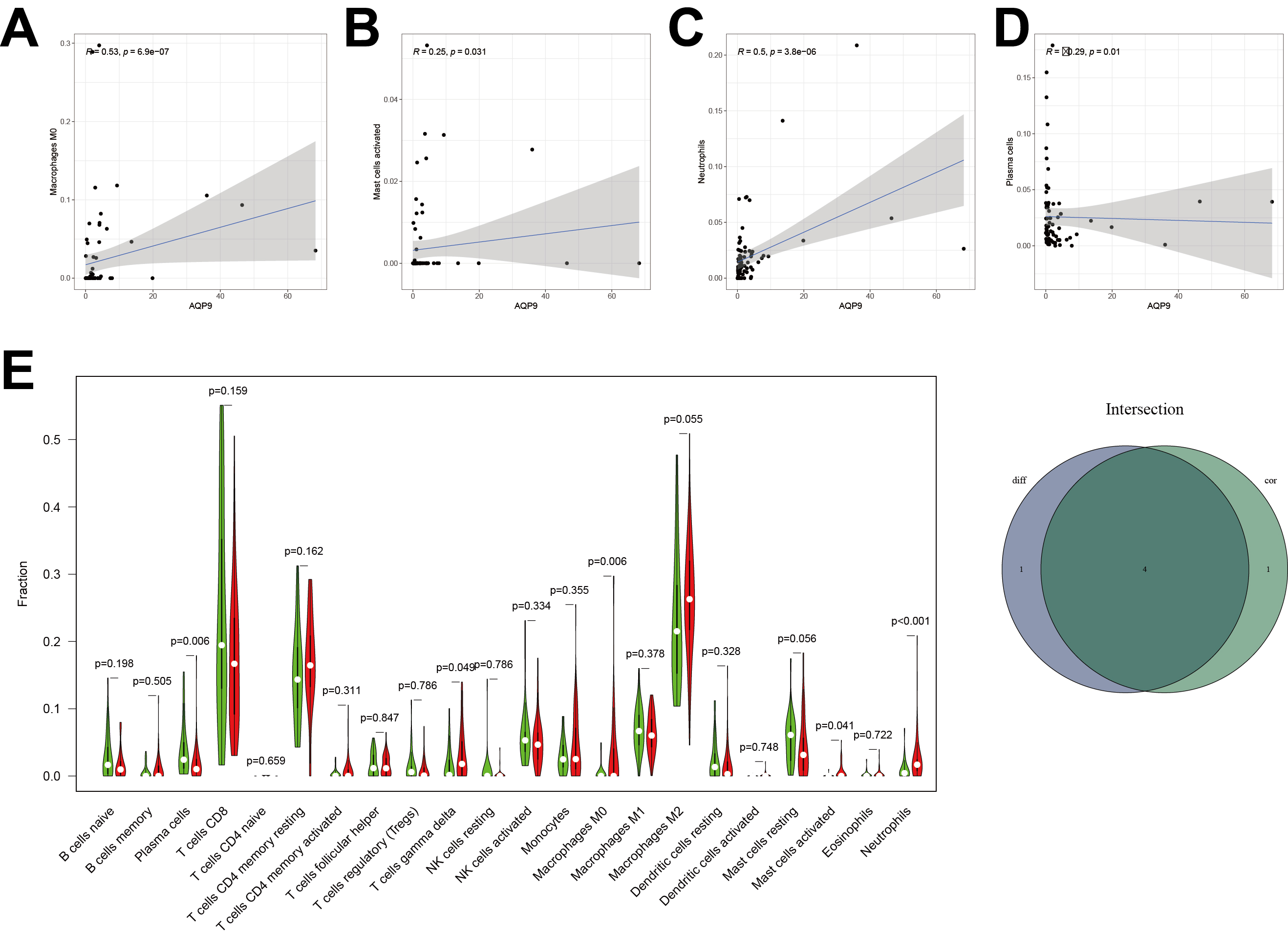

Supplement: Supplementary file 1 [file Image_1.jpeg]
